# Prospective blinded study of somatic mutation detection in cell-free DNA utilizing a targeted 54-gene next generation sequencing panel in metastatic solid tumor patients

**DOI:** 10.18632/oncotarget.5465

**Published:** 2015-10-05

**Authors:** Seung Tae Kim, Won Suk Lee, Richard B. Lanman, Stefanie Mortimer, Oliver A. Zill, Kyoung-Mee Kim, Kee Taek Jang, Seok-Hyung Kim, Se Hoon Park, Joon Oh Park, Young Suk Park, Ho Yeong Lim, Helmy Eltoukhy, Won Ki Kang, Woo Yong Lee, Hee-Cheol Kim, Keunchil Park, Jeeyun Lee, AmirAli Talasaz

**Affiliations:** ^1^ Division of Hematology-Oncology, Department of Medicine, Samsung Medical Center, Sungkyunkwan University School of Medicine, Seoul, Korea; ^2^ Department of Surgery, Gil Medical Center, Gachon University, School of Medicine, Incheon, Korea; ^3^ Guardant Health Inc., Redwood City, CA, USA; ^4^ The Innovative Cancer Medicine Institute, Samsung Medical Center, Seoul, Korea; ^5^ Department of Pathology and Translational Genomics, Samsung Medical Center, Sungkyunkwan University School of Medicine, Seoul, Korea; ^6^ Department of Surgery, Samsung Medical Center, Sungkyunkwan University School of Medicine, Seoul, Korea

**Keywords:** cell-free DNA (cfDNA), digital sequencing, genomic test

## Abstract

Sequencing of the mutant allele fraction of circulating cell-free DNA (cfDNA) derived from tumors is increasingly utilized to detect actionable genomic alterations in cancer.

We conducted a prospective blinded study of a comprehensive cfDNA sequencing panel with 54 cancer genes. To evaluate the concordance between cfDNA and tumor DNA (tDNA), sequencing results were compared between cfDNA from plasma and genomic tumor DNA (tDNA). Utilizing next generation digital sequencing technology (DST), we profiled approximately 78,000 bases encoding 512 complete exons in the targeted genes in cfDNA from plasma. Seventy-five patients were prospectively enrolled between February 2013 and March 2014, including 61 metastatic cancer patients and 14 clinical stage II CRC patients with matched plasma and tissue samples. Using the 54-gene panel, we detected at least one somatic mutation in 44 of 61 tDNA (72.1%) and 29 of 44 (65.9%) cfDNA. The overall concordance rate of cfDNA to tDNA was 85.9%, when all detected mutations were considered. We collected serial cfDNAs during cetuximab-based treatment in 2 metastatic KRAS wild-type CRC patients, one with acquired resistance and one with primary resistance. We demonstrate newly emerged *KRAS* mutation in cfDNA 1.5 months before radiologic progression. Another patient had a newly emerged *PIK3CA* H1047R mutation on cfDNA analysis at progression during cetuximab/irinotecan chemotherapy with gradual increase in allele frequency from 0.8 to 2.1%. This blinded, prospective study of a cfDNA sequencing showed high concordance to tDNA suggesting that the DST approach may be used as a non-invasive biopsy-free alternative to conventional sequencing using tumor biopsy.

## INTRODUCTION

Numerous molecularly targeted agents are now being developed for specific genomic aberrations, enabled by the efficient, rapid and accurate characterization of tumor genomes with next generation sequencing (NGS). For instance, non-small cell lung cancer (NSCLC) harboring *EGFR* mutations or *ALK* translocations and melanomas with *BRAF* mutations have been shown to be highly sensitive to the corresponding targeted kinase inhibition [1–3]. *RAS* mutations predict resistance to *EGFR* antibody therapy in colon cancer [4]. Subsequently, somatic mutation analysis of known or potential actionable oncogenes has now become part of the routine practice in medical oncology [5, 6]. As the number of genomic targets with matched therapies increases rapidly in the current oncology era, tissue biopsy material is now becoming an issue since genomic testing heavily relies on relatively small core or fine needle aspiration in metastatic patients [7, 8]. Until now, tumor tissue specimens have been the standard source of tumor DNA for clinical and research sequencing; however, acquisition of tumor tissue is not always feasible in patients with metastatic disease and may delay decision-making [9]. In addition, surgical or needle aspiration biopsy of visceral primary or metastatic tumors often are associated with significant medical costs and potential complications. Circulating blood biomarkers may constitute non-invasive real-time surrogates for diagnosis, prognosis, therapeutic tailoring, and resistance monitoring and mitigate needle biopsy sampling errors related to intra- or inter-tumor heterogeneity [10, 11]. For these reasons, sequencing of circulating cell-free DNA (cfDNA) has been suggested as a reasonable alternative to tumor tissue-based genomic testing [12–14].

In this study, we utilized a novel NGS panel of 54 clinically actionable genes utilizing digital sequencing of cell-free circulating tumor DNA isolated from a non-invasive blood draw (see Table S1 in the Supplementary). The test detects single nucleotide variants in all 54 genes and copy number amplifications in *EGFR*, *ERBB2* (HER2) and *MET* [15]. We evaluated the concordance in genomic alterations between paired plasma cfDNA and primary tumor DNA (tDNA) samples using the same NGS method. We then conducted a prospective blinded validation of the targeted cfDNA panel via an inter-laboratory comparison of key oncogenes identified with tumor tissue using direct DNA sequencing (*KRAS* and *BRAF*) or hotspot analysis (*KIT*) to a second laboratory performing digital sequencing of cfDNA in corresponding plasma samples, while keeping the latter blind to the PCR reference standard results. Lastly, we tested the use of cfDNA as a follow-up monitoring of potential evolving mutations during cetuximab-based chemotherapy in metastatic colorectal cancer patients in an exploratory analysis.

## RESULTS

### Patient characteristics

**Figure 1:**
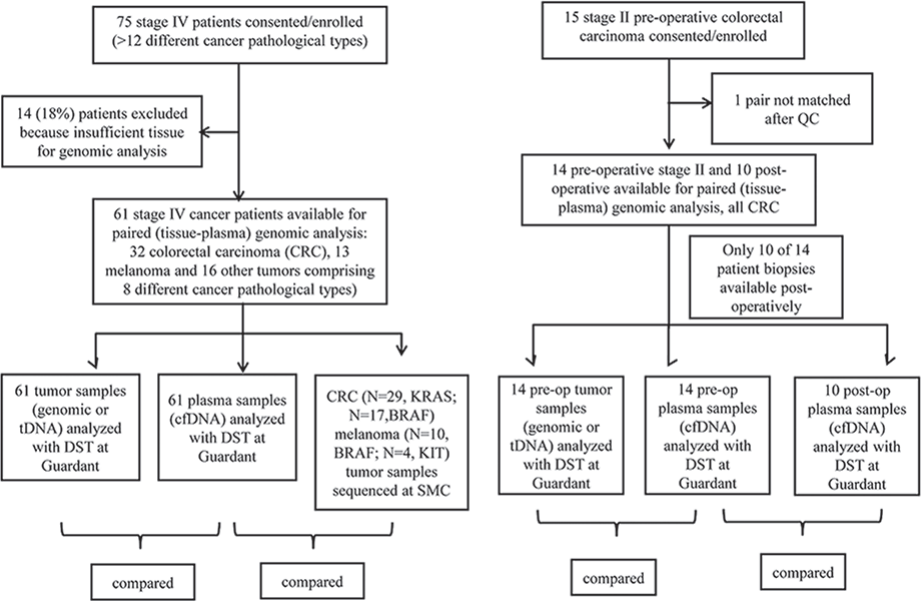
STARD diagram

Between February 2013 and March 2014, 75 advanced solid tumor patients were consented and enrolled in this study (clinicaltrials.gov, NCT02067754). Fourteen patients were excluded because of insufficient tissue for genomic analysis as illustrated in the STARD flowchart diagram [16] (Figure 1). Tumor section or biopsy of the primary tumor or metastasis and blood collection were conducted in all consented patients and the protocol was approved by the institutional review board. Table 1 provides the baseline patients characteristics. The most frequent cancer type was colorectal cancer (CRC) (*n* = 32, 52.6%), followed by melanoma (*n* = 13, 21.4%), gastrointestinal stromal tumor (GIST) (*n* = 4, 6.6%), renal cell carcinoma (RCC) (*n* = 3, 4.9%), gastric cancer (*n* = 3, 4.9%), sarcoma (*n* = 2, 3.2%), then 4 others with various cancer types. 87% of the patients had stage IV disease at the time of cfDNA analysis and most tDNAs (90.2%) were obtained from primary tumor sites. When dichotomized according to sampling interval between tumor tissue and blood sampling (synchronous sampling; sampling interval ≤ 6 months vs. metachronous sampling; sampling interval > 6 months), the majority of patients (71.9%) were in the synchronous sampling category. We included 14 clinical stage II colon cancer patients to compare primary tDNA and cfDNA to evaluate the concordance at the time of surgery, and also cfDNA 7-day post-surgery (10 patients) to detect the impact of surgical resection on cfDNA levels.

**Table 1:** Characteristics of metastatic cancer patients with genotyping analysis for paired tumor-tissue and cfDNA (*N* = 61)

Characteristic	Number	(%)
Age (years)		
Median (range)	57	(29–83)
Sex		
Male	39	(63.3)
Female	22	(37.3)
Disease types		
Colorectal cancer	32	52.6%
Melanoma	13	21.4%
Gastrointestinal stromal tumor (GIST)	4	6.6%
Renal	3	4.9%
Gastric	3	4.9%
Sarcoma	2	3.2%
Bladder	1	1.6%
Neuroendocrine tumor	1	1.6%
Pancreatic cancer	1	1.6%
Thyroid cancer	1	1.6%
Total	61	100%
Pathologic stage		
Stage IV	61	100%
No. of metastatic sites		
1	11	(18.0)
>=2	50	(82.0)
Tumor sample origin		
Primary sites	55	(90.2)
Metastasis	6	(9.8)
Sampling interval between Tumor tissue and Blood		
Synchronous (≤ 6 months)	34	(55.7)
Metachronous (> 6 months)	27	(44.3)

### Concordance between cfDNA and tDNA sequencing results

All 61 metastatic cancer patients with paired cfDNA and tDNA samples available were successfully sequenced with the DST method. For tDNA, a somatic mutation (clinically significant variants, and variants reported in COSMIC) was found in 44 samples (72.1%) while 17 samples (27.9%) had no significant genetic alterations in tDNA.

**Figure 2:**
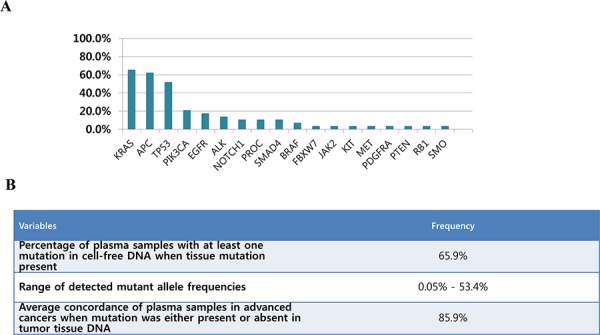
A. Mutational profiles (clinically significant variants, variants reported in COSMIC and other novel variants) detected in cfDNA for 61 advanced cancer patients with various tumor types and B. Details for genetic aberration analyzed in cfDNA and the concordance for comprehensive mutational profiles between tumor-tissue analysis and cfDNA

Figure 2A shows mutational profiles for cfDNA of 61 advanced cancer patients with various tumor types. For cfDNA, 29 samples (65.9%) had one or more somatic mutations with allele fractions ranging from 0.05%–53.4. The overall concordance rate between cfDNA and tDNA was 85.9%, when all detected mutations are considered (Figure 2B). Table 2 (see Table S2 in the Supplementary) shows mutational profiles of paired tumor tissue and cfDNA samples according to disease types.

**Table 2:** Two by two comparison tables for calculation of sensitivity, specificity and diagnostic accuracy for genomic aberrations in *KRAS* and *BRAF* between tumor tissues sequenced at Samsung Medical Center and blinded cfDNA analysis at Guardant Health for advanced colorectal cancer and *BRAF* for advanced melanoma patients Tumor-tissue next generation sequencing is used as the reference standard but in Table (A) for *KRAS* mutation status tumor-tissue NGS is used first as the reference standard then tissue NGS is compared to cfDNA NGS as the reference standard for comparison purposes

(A) Colorectal cancer						
*N* = 29	Tumor-tissue based reference standard analysis					
	*KRAS* (Exon 12, 13)	Mutant	WT	Sensitivity	Specificity	Accuracy
cfDNA NGS	Mutant	5	3	83.3%	86.9%	86.2%
	ND	1	20			
	Total	6	23			
*N* = 17	*BRAF V600E*	Mutant	WT	Sensitivity	Specificity	Accuracy
cfDNA NGS	Mutant	1	0	100%	100%	100%
	ND	0	16			
	Total	1	16			

(B) Melanoma						
*N* = 10	Tumor-tissue based reference standard analysis					
	*BRAF V600E*	Mutant	WT	Sensitivity	Specificity	Accuracy
cfDNA NGS	Mutant	4	0	100%	100%	100%
	ND	0	6			
	Total	4	6			
*N* = 4	**Tumor-tissue based reference standard analysis**					
	*KIT*	Mutant	WT	Sensitivity	Specificity	Accuracy
cfDNA NGS	Mutant	0	0	-	100%	100%
	ND	0	4			
	Total	0	4			

(WT = wild type in tissue, ND = Not Detected in cfDNA) (A) Colorectal cancer (B) Melanoma.

### Prospective inter-laboratory blinded comparison of index test (cfDNA) to reference test (direct DNA sequencing and hotspot analysis)

For the specific mutations used in the selection of matched therapy, we compared conventional direct DNA sequencing method (*KRAS*, *BRAF*, *KIT*) as the reference standard to cfDNA results using the Guardant360 DST panel. The DST team was completely blinded to the direct DNA sequencing results from SMC. For *KRAS* codons 12 and 13 mutations in 29 metastatic CRCs, 83.3% sensitivity, 86.9% specificity and 86.2% accuracy (Table 2A) were revealed between cfDNA and direct DNA sequencing of tumor FFPE specimens. For *BRAF* V600E mutation in 17 metastatic CRCs, sensitivity, specificity and accuracy were all 100% (Table 2B) between cfDNA and direct DNA sequencing of tumor FFPE specimens. In 10 melanoma patients, *BRAF* V600E mutation resulted in 100% sensitivity, specificity and accuracy between cfDNA and direct DNA sequencing of tumor FFPE specimens. *KIT* mutations detected in tDNA of 4 melanomas were also observed in the cfDNA DST panel.

### Monitoring of molecular resistance through sequencing assay using cfDNA sequencing

**Figure 3:**
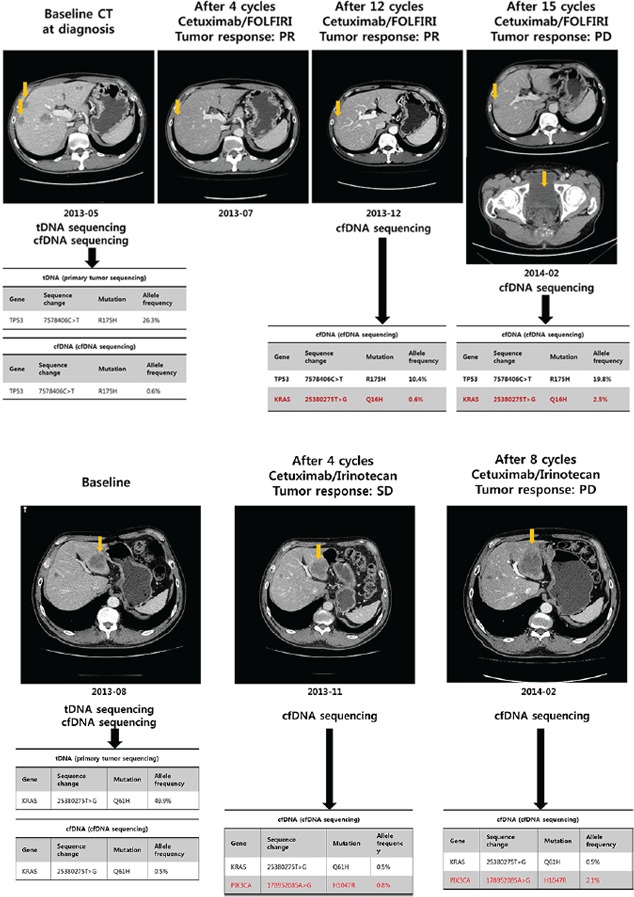
Patient monitoring with cfDNA during cetuximab-based treatment in metastatic colon cancer A. acquired resistance to cetuximab; B. primary resistance to cetuximab

A 55 year-old man was diagnosed with metastatic CRC at SMC. The major metastatic lesions were hepatic, and also involved abdominal and presacral lymph nodes. Before starting systemic chemotherapy, mutational profiles of both primary tumor tissue and plasma were evaluated. Both tumor DNA and cfDNA sequencing demonstrated *TP53* mutation and *KRAS* wild type (Figure 3A). cfDNAs were collected before cetuximab/FOLFIRI (5-FU/irinotecan/leucovorin) treatment, and at the time of computed tomography (CT) evaluation (every 4 cycles) thereafter. After 4 cycles of cetuximab/FOLFIRI chemotherapy, the follow-up CT scan revealed tumor shrinkage corresponding to partial response based on RECIST 1.1 criteria (Figure 3A). The patient continued to receive the same regimen for 6 months without definite radiologic or clinical progression. After 12 cycles of cetuximab/FOLFIRI, new *KRAS* mutation emerged in the patient's plasma cfDNA without evidence of radiologic progression. After 1.5 months from the time of newly emerged *KRAS* mutation emergence in cfDNA, the patient exhibited radiologic progression with sacral metastasis, showing persistence of the *KRAS* mutation in cfDNA (Figure 3A). Another patient with *KRAS* Q61H mutant on both tDNA and cfDNA had stable disease and a reduction in the cfDNA *KRAS* Q61H mutant allele frequency from 49.9% to 0.5% after four cycles of cetuximab/irinotecan (Figure 3B). However, this patient had a newly emerged *PIK3CA* H1047R mutation on cfDNA analysis at 2 months of cetuximab/irinotecan chemotherapy with allele frequency of 0.8%. Because the treatment response was within stable disease per RECIST 1.1 criteria, the patient received 4 more cycles of cetuximab/irinotecan. At 4 months CT evaluation (after 8 cycles), the patient had definite radiologic progression with enlarging liver metastases and parallel increase in allele frequency of PIK3CA H1047R mutation to 2.1%.

### Analysis of cfDNA in stage II colorectal cancer patients

Fourteen patients with clinical stage II colorectal cancer received surgical treatment. Blood samples before (day 0) and after surgery (post-operative day 7) were analyzed with the cfDNA DST. The concordance rate for cfDNA to tDNA was 90.0% (95% CI, 66.7% – 98.6%) (see Figure S1A in the Supplementary). Among the ten patients with post-operative day 7 plasma samples, eight patients showed a dramatic reduction in cfDNA (see Figure S1B and Table S3 in the Supplementary). The recurrence data is being collected for this patient cohort.

## DISCUSSION

In this prospective blinded study, the cfDNA DST panel revealed a very high sensitivity, specificity and accuracy compared with tissue-based reference standard analysis for *KRAS* and *BRAF* in CRC. Although the specificity for the DST for *KRAS* mutation in cfDNA compared to tissue sequencing as the reference standard analysis was 86.9%, it is likely that a cfDNA false positive reflects a tissue biopsy-based false negative, given that analytic specificity studies of DST against whole exome sequencing demonstrate near-perfect specificity. Considering DST of cfDNA as the gold standard, tissue biopsy-based *KRAS* mutation detection sensitivity as reference standard analysis was only 83.3%. The likely explanation for this is tumor intra- or inter-tumor heterogeneity not captured by core needle or surgical tissue biopsy sampling. However, cell-free DNA analysis did not detect the *KIT* mutations found in the six positive melanoma samples, perhaps reflecting that these tumors although at advanced stage, did not release tumor DNA into the circulation or false-negative results.

Analytic sensitivity for the cfDNA DST method via dilution studies has shown that a single DNA fragment with a somatic mutation can be detected in a background of 1,000 germline fragments (0.1% mutant allele fraction). Analytic specificity was also shown to be 99.9999% with a single false positive nucleotide result in nearly 1.6 million bases, covering the 54 genes in the panel (ref Lanman *et al*. Analytical and Clinical Validation of a Digital Sequencing Panel for Quantitative, Highly Accurate Evaluation of Cell-free Circulating Tumor DNA, submitted). The negligible false positive rate for such a long (78 kbp) targeted region is the differentiating feature of this comprehensive cfDNA assay, relative to other tumor sequencing assays which typically manage the false positive rate by sequencing short regions of a small number of hotspots or hot exons in a few genes.

In metastatic CRC, 35% of patients harbored *KRAS* exon 2 mutations and 12% of patients harbored *BRAF* exon 15 mutations. Currently, *KRAS* and *BRAF* mutations are routinely tested in tumor tissue by various methods for selection of anti-EGFR therapy in metastatic CRC patients [17–22]. Although these tissue-based methods have proven clinical utility, they depend on the availability of tumor samples, as well as quality and the quantity of the tumor specimen. Tissue specimens must first undergo pathologic review to assure adequate tumor cell content and this process plus the sequencing workflow itself may have a long data turnaround (2–3 weeks at SMC). In contrast, 100% of cfDNA samples were successfully sequenced within 10 days using DST despite the transport distance between Korea and California. It has been shown that patients with tumor mutations in *KRAS* exon 2 as well as *KRAS* exons 3 or 4 or *NRAS* exon 2, 3, or 4 are likely to show resistance to anti-EGFR agents [23]. Thus, in order to select only the wild type *RAS* CRC population for anti-EGFR treatment, more rapid and efficient methods of genomic assessment are needed. Comprehensive cfDNA analysis using sequencing assay with DST might be a useful candidate method that could meet these needs.

The emergence of clinical resistance to previously effective anti-neoplastic therapy results from the acquisition of molecular alterations in genes or pathways that govern the resistant mechanisms. Defining these mechanisms of resistance to targeted agents is difficult because it is extremely difficult to acquire serial tumor biopsies in patients with advanced disease at multiple time points. In this study, we demonstrated a potential clinical application of cfDNA genomics which may allow detection of emergence of genomic alterations at acquired resistance to targeted therapy. Although it is an exploratory analysis, we attempted to monitor serial changes of mutational profiles for cfDNA in a *KRAS* wild-type CRC patient receiving cetuximab-based chemotherapy. In this patient, we observed that the emergence of *KRAS* mutation was associated with secondary resistance to cetuximab-based chemotherapy. Detection of the *KRAS* variant in cfDNA of this patient was ascertained before radiologic relapse. This finding is consistent to those of previous studies [24, 25]. Nevertheless, DST allows sequencing of 54 genes rather than hotspot mutations in cfDNA which provide broader opportunities to detect newly emerged genomic alterations

There have been limited studies utilizing NGS technologies for the detection of tumor somatic mutations in body fluids [26–29]. Narayan *et al*. reported that a deep sequencing assessment can be a useful strategy for the detection of low abundance point mutations in surrogate tissues [30]. Our study, assessing mutations in a comprehensive panel of genes and performed on patients with a large variety of tumor types, further extends the applicability of the NGS for analysis of aberrant genomic events in cfDNA.

Analytic sensitivity should not be confused with clinical sensitivity, however. Although the DST panel has high analytic sensitivity when DNA from cell lines with known mutations is spiked into plasma, it is limited by biology, i.e. the assay cannot measure cfDNA in patients whose tumors do not shed significant DNA into the circulation, such as stage I cancers or primary brain tumors that are isolated from systemic circulation by the blood-brain barrier. These factors limit clinical sensitivity of any cfDNA method. Despite this potential limitation, the concordance rate for all mutations found across 54 driver genes in various tumor types was 85.9% between tDNA and cfDNA.

This is the first blinded, prospective, external validation study to compare NGS of a comprehensive 54-gene panel using matched cfDNA and tDNA samples. We demonstrate a high concordance rate between cfDNA and tDNA and showed that cfDNA can be utilized as a monitoring tool for newly emerged mutations. The concordance rate for all mutations found across 54 genes in solid tumors was 85.9% between tDNA and cfDNA. In an exploratory analysis, we monitored serial changes of mutational profiles for cfDNA in two *KRAS* wild-type CRC patients receiving cetuximab-based chemotherapy. Detection of the *KRAS* variant in cfDNA of this patient was detected before radiologic relapse. Our study suggests the potential utility of cfDNA cancer panel as an alternative genomic test obviating the need for tumor biopsy at diagnosis and at resistance. Currently, we are testing the impact of cfDNA in refractory cancer patients on progression-free survival in the NEXT-2 trial (NCT#02140463).

## MATERIALS AND METHODS

### Patients

The institutional review board of the Samsung Medical Center (SMC) approved the study. All study participants provided written informed consent before study entry. Briefly, consented patients with metastatic cancer eligible for clinical trial enrollment or chemotherapy based on genomic biomarkers were eligible to enter the study. Patients with pathologically confirmed cancer and who had either archived tissue or fresh tissues were eligible for genomic analysis. The STARD flow diagram for in this study was summarized in Figure 1 [15]. For stage II colorectal cancer patients, baseline blood for cfDNA at the time of surgery in the operation room and at postoperative 7-day follow-up sample were drawn. For this patient cohort, surgical specimens were procured at the time of surgery for tDNA analysis.

### Tumor samples

All tumor specimens (except for stage II colon) were paraffin embedded tumor tissues. Tumor areas (> 60%) were dissected under microscopy from 4-μm-thick unstained sections by comparison with an H&E stained slide, and genomic DNA was extracted using a Qiagen DNA FFPE Tissue Kit (Qiagen, Hilden, Germany) according to the manufacturer's instructions. After extraction, we measured concentrations and 260/280 and 260/230 nm ratios using a spectrophotometer (ND1000, Nanodrop Technologies, ThermoFisher Scientific, MA, USA). Each sample was then quantified with a Qubit fluorometer (Life Technologies, Carlsbad, CA, USA). Tumor tissue DNA (tDNA) was analyzed with direct DNA sequencing (*KRAS* and *BRAF*) and hotspot analysis (*KIT*) at SMC when quantity was sufficient. Simultaneously, at least 100ng of tumor DNA (tDNA) was sent for next-generation sequencing utilizing Digital Sequencing™ technology (as described below) at Guardant Health, Inc. (Guardant).

### Blood samples and circulating cell-free DNA isolation and quantification

For each enrolled patient, blood was collected during routine phlebotomy as part of standard cancer care. Blood samples were immediately processed upon receipt to isolate plasma. Plasma was isolated from EDTA tubes by centrifugation at 1,600 g during 10 minutes at 4°C. Plasma was aliquoted and stored at −70°C. CfDNA was extracted from aliquots (1 mL) of plasma using the QIAamp circulating nucleic acid kit (Qiagen) with the QIAvac 24 Plus vacuum manifold, following the manufacturer's instructions, and quantified by Qubit fluorometer (Life Technologies, Carlsbad, CA, USA). All cfDNA analysis was performed at Guardant.

### Digital sequencing technology (see Method in the Supplementary)

#### DNA sequencing for *KRAS* and *BRAF* and hotspot analysis (*KIT*)

*KRAS* mutation tests were performed at the designated central laboratory of SMC as described previously [31]. Mutations in codons 12 and 13 of the *KRAS* gene were detected by direct sequencing of polymerase chain reaction (PCR) products amplified from DNA extracted from representative tumor tissue. *BRAF* V600E direct sequencing and *KIT* hotspot mutations were tested according to our previous work [32]. Briefly, tumor-rich areas (>80%) were extracted from paraffin–embedded tissue sections, and 10 4-μm-thick sections containing a representative portion of each tumor block were subjected to DNA isolation using the QIAamp DNA Mini Kit (Qiagen, Hilden, Germany). Deeply pigmented samples were incubated with Chelex-100 (Bio-Rad Laboratories) to prevent PCR inhibition by melanin [33]. Purified DNA was incubated for 10 min at room temperature with an equal volume of a 5% Chelex-100 solution equilibrated in Qiagen AE buffer, heated to 95°C for 2 min, and allowed to cool. The Chelex-100 resin was pelleted in a microfuge, and the supernatant DNA used for PCR reactions. PCR products were processed for the DNA sequencing reaction using the ABI-PRISM BigDye Terminator version 3.1 (Applied Biosystems, Foster, CA, USA) with both forward and reverse sequence-specific primers. Sequence data were generated using the ABI PRISM 3100 DNA Analyzer (Applied Biosystems).

### Statistics

First, sequencing of plasma cfDNA was compared to sequencing of tDNA, and the concordance rate between sequencing of cfDNA and tDNA was defined as the percentage agreement for all mutations found in a patient when both cfDNA and tDNA were both positive for mutations or both negative (tissue wild type or cfDNA “not detected”), then averaged for all 61 patients with matched plasma-tissue sample pairs. Secondly, clinical validity was evaluated for specific key oncogenic mutations according to tumor types such as *KRAS* and/or *BRAF* in colorectal cancer, and *BRAF or KIT* in melanoma. Sensitivity, specificity and diagnostic accuracy were calculated by comparing sequencing results for cfDNA to direct DNA sequencing (*KRAS* and *BRAF*) and hotspot analysis (*KIT*) performed at our laboratory (SMC) [34]. The Guardant Health laboratory remained blinded to the SMC reference standard results for these key oncogenic mutations and SMC conducted the unblinding and statistical analysis of results.

## SUPPLEMENTARY METHODS FIGURE AND TABLES


