# Supplementary material for: Prospective blinded study of somatic mutation detection in cell-free DNA utilizing a targeted 54-gene next generation sequencing panel in metastatic solid tumor patients
Source: Oncotarget. 2015 Oct 5;6(37):40360–9. doi: 10.18632/oncotarget.5465 (PMC4741900; doi:10.18632/oncotarget.5465)
Supplement: Supplementary file 1 [file oncotarget-06-40360-s001.pdf]

SUPPLEMENTARY METHODS

Digital sequencing technology (see Method in the supplementary)

The Guardant360 Digital Sequencing technology (DST) enables high quality sequencing of each individual circulating DNA fragment in plasma with single molecule sensitivity. At the time of this analysis, the Guardant360 panel sequences 54 target onco-/tumor-suppressor genes including the complete exonic bases of 18 actionable cancer-related genes and the critical; ("hot") exons of an additional 36 genes (see Table 1 in the Supplement) (e.g., exons containing at least one or more reported somatic mutations in COSMIC or separate publications), resulting in a concatenated targeted region of approximately 78,000 base pairs (78 kbp). The test simultaneously sequences the 54 cancer-related genes to an average depth of coverage of 8,000X. Standard next-generation sequencing workflows are plagued by high noise (false positives) when mutant allele fraction (MAF) approach 5–10% or lower. This challenges analysis of tumor DNA in circulation where MAF across a range of advanced cancers were found to be as low as 0.1% (median 0.5%, interquartile range 0.2% - 2.5%). Since the numbers of false positives increase as the targeted region increases, most ctDNA assays manage the false positive rate by limiting the sequenced region to short read lengths covering only a few hotspots or hot exons. In contrast, DST technology enables nearly-perfect specificity, essentially eliminating the false positives encountered at very low ctDNA concentrations with standard sequencing workflows, even with the long 78 kbp targeted region required to cover 512 exons in the 54 gene panel. The DST workflow enables high-quality single molecule sequencing as the vast majority of input DNA molecules are converted to digital sequencing libraries regardless of length or content with input amounts ranging from 2–60 ng. Paired end sequencing by synthesis is performed on an Illumina Hi-Seq 2500, followed by proprietary algorithmic reconstruction of the digitized sequencing signals as previously described (ref Lanman *et al.*, submitted). The Guardant360 panel is an advanced laboratory diagnostic test (ADLT) offered by a sole source Clinical Laboratory Improvement Amendments (CLIA) licensed and College of American Pathologists (CAP) accredited clinical laboratory (Redwood City, California, USA). Genomic DNA isolated from tissue biopsies (tDNA) and paired plasma samples were analyzed at Guardant Health, Inc. for sequencing. In addition, plasma samples from 14 pre-operative stage II colorectal cancer patients, and 10 post-operative samples from the same set of patients, were sequenced at Guardant Health, Inc.

SUPPLEMENTARY FIGURE AND TABLES

A

Variables	
Number of Samples	14
Percent of samples analyzed with at least 1 alteration	61.5%
Concordance between tDNA and cfDNA	90.0%
Level of cfDNA pre-surgery	0.0-31.8%
Level of cfDNA post-surgery	0.0-0.4%

B

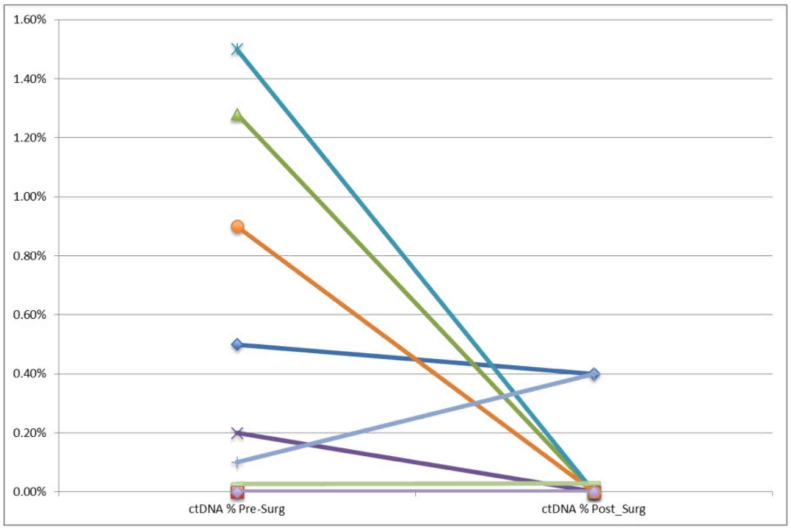

Supplementary Figure S1: Analysis of cfDNA in stage II colorectal cancer. A. Overall summary of analysis for tDNA and cfDNA in these patients **B.** Changes of cfDNA between pre-surgery (immediately before surgery) and post-surgery (day 7 after surgery) in 10 patients.

Supplementary Table S1: Comprehensive sequencing panel of 54 genes utilizing circulating cell-free DNA in plasma

Genes with all exons completely sequenced (18)

ALK	*APC*	*AR*	*BRAF*
CDKN2A	*EGFR*	*ERBB2*	*FBXW7*
KRAS	*MET*	*MYC*	*NOTCH1*
NRAS	*PIK3CA*	*PTEN*	*PROC*
RB1	*TP53*		

Genes with those exons with reported mutations completely sequenced (36)

ABL1	*AKT1*	*ATM*	*CDH1*	*CSF1R*	*CTNNB1*
ERBB4	*EZH2*	*FGFR1*	*FGFR2*	*FGFR3*	*FLT3*
GNA11	*GNAQ*	*GNAS*	*HNF1A*	*HRAS*	*IDH1*
IDH2	*JAK2*	*JAK3*	*KDR*	*KIT*	*MLH1*
MPL	*NPM1*	*PDGFRA*	*PTPN11*	*RET*	*SMAD4*
SMARCB1	*SMO*	*SRC*	*STK11*	*TERT**	*VHL*

* includes promoter region of TERT

Supplementary Table S2: Mutation profiles (clinically significant variants or variants reported in COSMIC) based on next generation sequencing of paired tumor tissue and cfDNA samples according to disease types (*N* = 61)

Disease type	*N*	Tumor tissue	cfDNA
Colorectal cancer (32)	1	*TP53*	*TP53*
	1	*KRAS, TP53, APC-R232*, APC-R216**	*KRAS, TP53, APC-R232*, APC-R216**
	1	*TP53, CTNNB1, FBXW7*	*TP53, CTNNB1*
	1	*KRAS, APC*	*TP53*
	1	*wild type*	*wild type*
	1	*TP53, APC*	*wild type*
	1	*APC*	*wild type*
	1	*APC, KRAS, TP53*	*APC, KRAS, TP53*
	1	*CTNNB1, KRAS, TP53*	*CTNNB1, KRAS, TP53*
	1	*TP53, APC*	*TP53, APC*
	1	*PIK3CA, APC, TP53, KRAS*	*PIK3CA, APC, TP53, KRAS*
	1	*wild type*	*wild type*
	1	*APC-E1374*, APC-R232*, ERBB2*	*APC-E1374**
	1	*KRAS, TP53, FBXW7-R578*, FBXW7-R425H,APC-R1114*, APC-E1309*,APC-R1920*, APC-R2204**	*wild type*
	1	*APC, TP53*	*APC, TP53*

(*Continued*)

Disease type	*N*	Tumor tissue	cfDNA
	1	*wild type*	*wild type*
	1	*ALK, TP53-C135Y*	*TP53-C135Y, TP53-S127P*
	1	*AKT1*	*wild type*
	1	*FBXW7, KRAS, PIK3CA, TP53, APC*	*FBXW7, KRAS, PIK3CA, TP53, APC*
	1	*KRAS, TP53, APC-S1282*, APC-R213**	*KRAS, TP53, APC-S1282*,APC-R213**
	1	*TP53, FBXW7, APC*	*TP53, FBXW7, APC*
	1	*wild type*	*wild type*
	1	*wild type*	*wild type*
	1	*APC, TP53-R110P*	*CTNNB1, TP53-R248Q*
	1	*BRAF*	*wild type*
	1	*NRAS, PTEN, TP53*	*wild type*
	1	*PIK3CA, APC, BRAF*	*wild type*
	1	*KRAS, TP53*	*wild type*
	1	*TP53, BRAF, ATM*	*TP53, BRAF, ATM*
	1	*KRAS, TP53, APC*	*KRAS, TP53*
	1	*KRAS, TP53*	*KRAS*
	1	*TP53, KRAS, APC*	*TP53, KRAS, APC*
Melanoma (13)	1	*wild type*	*wild type*
	1	*TP53, BRAF*	*wild type*
	1	*BRAF*	*BRAF, PIK3CA*
	1	*BRAF, CDKN2A*	*wild type*
	1	*BRAF*	*PIK3CA, BRAF*
	1	*BRAF*	*BRAF*
	1	*wild type*	*TP53*
	1	*NRAS*	*EZH2, NRAS*
	1	*BRAF*	*BRAF*
	1	*KRAS*	*wild type*
	1	*MET*	*MET*
	1	*FBXW7, PTEN, NRAS, PIK3CA*	*FBXW7, PTEN, NRAS*
	1	*TP53, BRAF*	*wild type*
GIST (4)	3	*wild type*	*wild type*
	1	*wild type*	*KIT*
Renal cell carcinoma (3)	1	*wild type*	*wild type*
	1	*VHL*	*wild type*
		VHL, GNAS	*GNAS*
Gastric (3)	1	*wild type*	*wild type*

(*Continued*)

Disease type	*N*	Tumor tissue	cfDNA
	1	*SMAD4, GNAS*	*wild type*
	1	*TP53*	*wild type*
Sarcoma (2)	2	*wild type*	*wild type*
Bladder (1)	1	*TP53-N239D*	*TP53-V274A*
Neuroendocrine (1)	1	*wild type*	*HRAS*
Pancreatic cancer (1)	1	*KRAS, GNAS*	*KRAS, GNAS*
Thyroid cancer (1)	1	*BRAF*	*KRAS, CDKN2A,BRAF*

Supplementary Table S3: In paired samples of 14 Stage II colorectal carcinoma patients, eight (61.5%) of the cfDNA samples contained SNVs out of the 13 positive tissue samples

Sample #	cfDNA SNVs	tDNA SNVs	Concordance (plasma to tissue)
1	*PIK3CA, APC, BRAF*	ND	0%
2	*APC*	ND	0%
3	*KRAS, TP53*	ND	0%
4	*PIK3CA, FBXW7, KRAS*	*PIK3CA, FBXW7, KRAS*	100%
5	*APC, TP53*	*APC, TP53*	100%
6	*TP53*	*TP53*	100%
7	*KRAS*	*KRAS*	100%
8	*TP53*	*TP53*	100%
9	*APC, TP53*	*APC, TP53*	100%
10	*PIK3CA, APC, KRAS, TP53*	*PIK3CA, APC, KRAS, TP53*	100%
11	*APC, TP53-G245S*	*APC, TP53-G245S, TP53-Y163C*	100%
12	*APC*	*TP53, ERBB2*	0%
13	*KRAS, APC*	ND	0%
Total			90.0% (95% CI, 66.7% - 98.6%)

Concordance in samples where both samples were positive or both negative for mutations was 90% (includes a 14th patient whose tissue was wild type and cfDNA was “not detected” (ND)).
